# miRNA-Mediated Knockdown of ATXN3 Alleviates Molecular Disease Hallmarks in a Mouse Model for Spinocerebellar Ataxia Type 3

**DOI:** 10.1089/nat.2021.0020

**Published:** 2022-06-01

**Authors:** Rui Jorge Nobre, Diana D. Lobo, Carina Henriques, Sonia P. Duarte, Sara M. Lopes, Ana C. Silva, Miguel M. Lopes, Fanny Mariet, Lukas K. Schwarz, M.S. Baatje, Valerie Ferreira, Astrid Vallès, Luis Pereira de Almeida, Melvin M. Evers, Lodewijk J.A. Toonen

**Affiliations:** ^1^ Center for Neuroscience and Cell Biology (CNC), Molecular Therapy of Brain Disorders Group, University of Coimbra, Coimbra, Portugal.; ^2^ Center for Innovative Biomedicine and Biotechnology (CIBB), Vectors, Gene and Cell Therapy Group, University of Coimbra, Coimbra, Portugal.; ^3^ ViraVector–Viral Vector for Gene Transfer Core Facility and University of Coimbra, Coimbra, Portugal.; ^4^ Institute for Interdisciplinary Research (III), University of Coimbra, Coimbra, Portugal.; ^5^ uniQure Biopharma b.v., Amsterdam, The Netherlands.; ^6^ Faculty of Pharmacy, University of Coimbra, Coimbra, Portugal.

**Keywords:** spinocerebellar ataxia type 3, AAV, gene therapy, miRNA, ataxin-3, ATXN3

## Abstract

Spinocerebellar ataxia type 3 (SCA3) is a neurodegenerative disorder caused by the expansion of a CAG repeat in the *ATXN3* gene. This mutation leads to a toxic gain of function of the ataxin-3 protein, resulting in neuronal dysfunction and atrophy of specific brain regions over time. As ataxin-3 is a dispensable protein in rodents, ataxin-3 knockdown by gene therapy may be a powerful approach for the treatment of SCA3. In this study, we tested the feasibility of an adeno-associated viral (AAV) vector carrying a previously described artificial microRNA against *ATXN3* in a striatal mouse model of SCA3. Striatal injection of the AAV resulted in good distribution throughout the striatum, with strong dose-dependent ataxin-3 knockdown. The hallmark intracellular ataxin-3 inclusions were almost completely alleviated by the microRNA-induced *ATXN3* knockdown. In addition, the striatal lesion of dopamine- and cAMP-regulated neuronal phosphoprotein (DARPP-32) in the SCA3 mice was rescued by *ATXN3* knockdown, indicating functional rescue of neuronal signaling and health upon AAV treatment. Together, these data suggest that microRNA-induced ataxin-3 knockdown is a promising therapeutic strategy in the treatment of SCA3.

## Introduction

Spinocerebellar ataxia type 3 (SCA3) is an autosomal-dominant neurodegenerative disorder belonging to the polyglutamine disorders. SCA3 is the most common polyglutamine SCA, with a prevalence estimated between 1 and 2 per 100,000 individuals, although this number differs per country [1,2].

Disease symptoms can vary substantially, but usually manifest around midlife and generally correlate with the mutational expansion of the gene [3]. SCA3 presents clinically heterogeneous, but the main symptom is a progressive cerebellar ataxia that affects balance, gait, and speech. Other symptoms include pyramidal signs, external ophthalmoplegia, dysarthria, rigidity, distal muscle atrophy, and double vision [4,5]. These symptoms are the result of a progressive neuronal loss in the cerebellum, thalamus, midbrain, pons, medulla, and spinal cord [6]. Patients ultimately die around 15 years after symptom onset as a result of loss of brainstem-related functions [7]. At present, there is no treatment available that can halt disease progression, and only symptomatic relief can be offered.

The cause of SCA3 is the expansion of a CAG repeat in exon 10 of the *ATXN3* gene, located on chromosome 14q32.1 [8]. In healthy individuals, a repeat range up to 40 CAGs is observed, whereas affected individuals present with repeat ranges between 54 and 89 [9]. The CAG repeat can explain ∼50% of variability in age of SCA3 onset [10], and patients with two expanded alleles develop a more severe disease phenotype [11]. The expanded repeat in the *ATXN3* gene is translated into a corresponding expanded polyglutamine repeat in the C-terminal region of the ataxin-3 protein. This in turn conveys a toxic gain of function to the protein, as well as misfolding and aggregation, which can potentially interfere with cellular homeostasis [12–14]. The characteristic nuclear accumulation of the polyglutamine-containing protein fragments is a hallmark of all polyglutamine diseases, although direct involvement in cellular toxicity is unclear.

Ataxin-3 normally functions as a deubiquitinating enzyme, able to bind and cleave specific ubiquitin chains present on other proteins [15,16]. In this manner, ataxin-3 is involved in regulating the proteasomal protein degradation [17], but has also been described to have activity as a transcriptional regulator [18] and is involved in the DNA damage response [19].

Despite the protein functions ascribed to ataxin-3, it is seemingly dispensable, at least in rodents, where knockout mice are viable, fertile, have a normal life span, and do not present gross abnormalities apart from slight increase in protein ubiquitination levels and an increase in anxiety [20,21]. Silencing ataxin-3 on human and mouse cell lines resulted in an accumulation of ubiquitinated proteins, defects on cytoskeleton/adhesion, and a marked increase in cell death [22]. Pathological mechanisms of SCA3 and the other polyglutamine disorders have proven complex, and much still remains to be discovered regarding the toxic pathways involved. In recent years, the involvement of repeat-associated non-ATG translation as well as RNA toxicity from the expanded *ATXN3* transcript has for instance been suggested [13], as well as involvement of DNA repair deficiency [23].

The monogenetic nature and well-established mutational profile make SCA3 a good candidate for gene therapies [24]. Indeed, efforts are ongoing to downregulate the ataxin-3 protein using either antisense oligonucleotides [25–27] or shRNA and siRNAs [28–35]. The main advantage of these strategies is that the most upstream cause of toxicity, the mutant *ATXN3* transcript, is directly inhibited. All toxic cellular pathways contributing to SCA3 pathology are hence thought to be prevented by *ATXN3* downregulation, which in turn should halt disease progression. Antisense oligonucleotides or RNAi-mediated targeting of transcripts each have their own advantages with respect to frequency of treatment, targeting of particular tissues, and the possibility to stop treatment [36].

Recently, we have developed and tested an artificial miRNA capable of downregulating ataxin-3 [37]. The miRNA is expressed from an expression vector and is processed through a noncanonical, dicer-independent pathway [38]. Although expressed from a naturally occurring miRNA scaffold, the miRNA used here is designed to be fully complementary to the human *ATXN3* sequence. The miRNA thus exerts its effect through the RISC-mediated RNA cleavage pathway, rather than translational repression associated with incomplete binding to untranslated regions of genes [39].

The miRNA used here, termed miATXN3, is delivered by means of an adeno-associated virus (AAV) serotype 5, and upon expression specifically and efficiently knocked down both ataxin-3 alleles [37]. Injection of AAV5-miATXN3 in cisterna magna of a transgenic knock-in mouse resulted in vector uptake in brainstem, cerebellum, and cortex.

Ataxin-3 protein knockdown in the brainstem and cerebellum of injected mice was up to 50%. In this mouse model, however, no pathological molecular phenotype could be detected. For this reason, we here tested AAV5-miATXN3 in a second, striatal lentiviral-based mouse model of SCA3.

This model is generated by injecting a lentiviral vector encoding a human mutant ataxin-3 with 72 glutamines (LV-mutATXN3) as described previously [40–42]. Expression of this construct results in the formation of characteristic ataxin-3 aggregates and neuropathological changes at the site of injection. Upon coinjection of LV-mutATXN3 and AAV5-miATXN3, a strong reduction in the mutant ataxin-3 expression and corresponding aggregates were observed. Neuronal damage was also almost completely abolished, showing that preventing expression of mutant *ATXN3* expression by AAV5-miATXN3 can alleviate the ataxin-3-induced neurotoxicity.

## Materials and Methods

### DNA expression constructs

The miATXN3 microRNA expression construct used here was generated in previous research by Martier *et al.*, where a pol II promoter drives expression of the construct carrying a miR-451 scaffold. The miRNA sequences and AAV5 vector production are described in [37].

A lentiviral vector containing the full-length human ataxin-3 coding sequence with 72 glutamines under control of phosphoglycerate kinase promoter was generated as previously described [40,43].

### Striatal viral injections in mice

All experimental protocols with mice were approved by ORBEA (Órgão Responsável pelo Bem-Estar dos Animais da Faculdade de Medicina da Universidade de Coimbra e do Centro de Neurociências e Biologia Celular) and performed in accordance with the European Community directive (2010/63/EU) covering the protection of animals used for scientific purposes. All researchers of this study received proper training (FELASA-certified course) and certification from the Portuguese authorities (Direcção Geral de Alimentação e Veterinária).

Injections were performed as described previously [44]. In brief, C57/Bl6 wild-type mice of 2 months of age were stereotaxically injected in the left and right striatum with a mixture of lentiviral vectors encoding mutant ataxin-3 with 72 glutamines (LV-mutATXN3) and PBS 1 × or AAV5-miATXN3 (three doses). The AAV5-miATXN3 used here (miATXN3_9) was produced [45] and selected [37] using previously described methods.

The injection coordinates in mouse brain were as follows: anteroposterior: +0.6 mm; lateral: ±1.8 mm; ventral: −3.3 mm; and tooth bar: 0. These coordinates correspond to the internal capsule, a large fiber tract passing through the middle of the striatum dividing both dorso-ventral and medial-lateral structures. Mice received 1.35 μL injections consisting of 0.35 μL of LV-mutATXN3 (400 ng of p24) and 1 μL of AAV5-miATXN3 [2 × 10^9^, 1 × 10^10^, or 5 × 10^10^ genome copies (gc)] in each hemisphere. Fifty-two days after injection, mice were killed for immunohistochemical analysis of morphological and neurochemical changes, as well as analysis of ataxin-3 mRNA and protein levels in the striatum.

For AAV5-GFP-injected mice, 1.3 × 10^11^ gc of AAV5-GFP in a volume of 2 μL was injected in the right striatum of wild-type mice of 6–8 weeks of age. Mice were killed 4 weeks after injection of AAV5-GFP for brain clearing and immunofluorescent imaging.

### Tissue preparation

After an overdose of ketamine/xilazine, mice were intracardiacally perfused with cold PBS 1 × . The brains were then removed and left and right hemispheres were divided. The right hemisphere was postfixed in 4% paraformaldehyde for 72 h at 4°C and cryoprotected by incubation in 25% sucrose/PBS 1 × for 48 h at 4°C. In the left hemisphere, the striatum was dissected and kept at −80°C for RNA/DNA/protein extraction. For each animal, 120 coronal sections of 25 μm were cut throughout the right brain hemisphere using a cryostat (LEICA CM3050S; Germany) at −20°C. Individual sections were then collected and stored in 48-well plates, as free-floating sections in PBS 1 × supplemented with 0.05% sodium azide at 4°C.

### Purification of total RNA and protein from mouse striata

Left part of the striatum was grinded with pellet pestles and homogenized with QIAshredder (Qiagen) columns. After homogenization, RNA, DNA, and protein were isolated using All Prep DNA/RNA/Protein Kit (Qiagen) according to the manufacturer's instructions. The initial volume of buffer RLT added to the striatum was 350 μL. Total amount of RNA was quantified using a Nanodrop 2000 Spectrophotometer (Thermo Scientific) and the purity was evaluated by measuring the ratio of OD at 260 and 280 nm. Protein was dissolved in a solution of 8 M urea in 100 mM Tris-HCl pH8 1% SDS and sonicated at 50 mA with 1 pulse of 3 s. Total RNA and protein extracts were stored at −80°C.

### Vector genome copies determination

The concentration of total DNA was determined by using NanoDrop 2000 spectrophotometer. Primers and probe specific for polyA tail of the vector construct sequence were used to measure the vector genome copies using TaqMan Fast Virus 1-Step Master Mix (Applied Biosystems; 4444464) and TaqMan Exogeneous Internal Positive Control (Applied Biosystems; 4308323). The vector genome copies were determined by quantitative real-time PCR (qPCR) through interpolation of a standard line of the expression cassette, and reported per microgram of genomic DNA input of the samples.

### Determination of MicroRNA expression levels

Mature miATXN3 microRNA levels were assayed using a custom TaqMan assay based on an automated design tool (Thermo Fisher Scientific; assay ID: CTEPRZE). The TaqMan MicroRNA reverse transcription kit (Applied Biosystems; 4366597) was used to generate cDNA. The microRNA expression level was determined by TaqMan Fast Universal Master Mix (Applied Biosystems; 4352042) with the Custom TaqMan small RNA assay for miATXN3. The level of molecules of miATXN3 by qPCR per microgram of total RNA input of the samples was calculated by interpolation of a standard line of the miATXN3 transgene.

### cDNA synthesis and qPCR

First, to avoid genomic DNA contamination in RNA preps, DNase treatment was performed using Qiagen RNase-Free DNase Set (Qiagen, Hilden, Germany), according to the manufacturer's instructions. cDNA was then obtained by conversion of total decontaminated RNA using the iScript Select cDNA Synthesis Kit (Bio-Rad, Hercules, CA) according to the manufacturer's instructions. After reverse transcriptase reaction, the mixtures were stored at −20°C.

Quantitative real-time PCR was performed using the SsoAdvanced SYBR Green Supermix (BioRad), according to the manufacturer's instructions. In brief, the qPCR was performed in a total volume of 20 μL, containing 10 μL of this mix, 10 ng of DNA template, and 500 nM of validated specific forward and reverse primers for human ataxin-3 (5′-TCCAACAGATGCATCGACCA-3′ and 5′-ACATTCGTTCCAGGTCTGTT-3′), mouse ataxin-3 (5′-GCAGATGATCAAGGTCCAACAG and 5′-TGAGGGCACTCTGCTCTTTC-3′), and mouse hypoxanthine guanine phosphoribosyl transferase (HPRT) (5′-CTTCCTCCTCAGACCGCTTT-3′ and 5′-TCATCGCTAATCACGACGCT-3′).

The qPCR protocol was initiated by a denaturation program (95°C for 30 s), followed by 40 cycles of two steps: denaturation at 95°C for 5 s and annealing/extension at 56°C for 10 s. The cycle threshold (Ct) values were determined automatically by the StepOnePlus software (Life Technologies). For each pair of primers, quantitative PCR efficiency was previously determined. The mRNA relative quantification with respect to control samples was determined by the Pfaffl method [46]. A control RNA sample lacking reverse transcriptase in the cDNA synthesis reaction was taken along in the qPCR to confirm DNAse treatment efficacy and exclude vector DNA detection.

### Western blotting

BCA protein assay kit (Thermo Fisher Scientific) was used to determine protein concentration. Seventy micrograms of striatum protein extracts were resolved on sodium dodecyl sulfate–polyacrylamide gels (4% stacking and 10% running). Proteins were then transferred onto a polyvinylidene difluoride membrane (Millipore), blocked with 5% nonfat milk powder dissolved in 0.1% Tween-20 in Tris-buffered saline for 1 h at room temperature. Membranes were then incubated overnight at 4°C with primary antibodies: mouse anti-1H9 (1:1000; Millipore) and mouse anti-β-actin (1:5000). The correspondent alkaline phosphatase-linked goat anti-mouse secondary antibody was incubated for 2 h at room temperature.

Bands were detected after incubation with enhanced chemifluorescence substrate (GE Healthcare) and visualized in chemiluminescent imaging (ChemiDoc™ Touch Imaging System; Bio-Rad Laboratories). Semiquantitative analysis was carried out based on the bands of scanned membranes using ImageJ software (National Institutes of Health) and normalized with respect to the total amount of protein loaded in the corresponding lane of the same gel.

### Immunohistochemistry

For each animal, 16 and 12 coronal sections with an intersection distance of 200 μm were selected for dopamine and cAMP-regulated neuronal phosphoprotein (DARPP-32) and 1H9 (ataxin-3) staining, respectively.

The procedure started with endogenous peroxidase inhibition by incubating the sections in PBS containing 0.1% phenyl hydrazine (Merck), for 30 min at 37°C. Subsequently, tissue blocking and permeabilization were performed in 0.1% Triton X-100 with 10% NGS (normal goat serum; Gibco) prepared in PBS, for 1 h at room temperature. Sections were then incubated overnight at 4°C with the primary antibodies rabbit anti-DARPP-32 (Millipore) and chicken anti-1H9 (HenBiotech), previously prepared on blocking solution at the appropriate dilution (1:2000). After three washings, brain slices were incubated in anti-rabbit or anti-chicken biotinylated secondary antibody (Vector Laboratories) diluted in blocking solution (1:250), at room temperature for 2 h.

Subsequently, free-floating sections were rinsed and treated with Vectastain ABC kit (Vector Laboratories) during 30 min at room temperature, inducing the formation of avidin/biotinylated peroxidase complexes. The signal was then developed by incubating slices with the peroxidase substrate: 3,3′-diaminobenzidine tetrahydrochloride (DAB Substrate Kit; Vector Laboratories). The reaction was stopped after achieving optimal staining, by washing the sections in PBS. Brain sections were subsequently mounted on gelatin-coated slides, dehydrated in an ascending ethanol series (75%, 95%, and 100%), cleared with xylene, and finally coverslipped using Eukitt mounting medium (Sigma-Aldrich).

### Evaluation of the volume of DARPP-32-depleted region

Images of coronal brain sections subjected to immunohistochemistry were obtained in Zeiss Axio Scan.Z1 microscope. Whole-brain images were acquired with a Plan Apochromat 20 × /0.8 objective. The extent of DARPP-32 loss in the striatum was analyzed by digitizing the stained sections (25 μm thickness sections at 200 μm intervals) to obtain complete rostrocaudal sampling of the striatum. To calculate the area of DARPP-32 loss, sections were analyzed using the Zen software (Zeiss). The depleted volume of the striatum was estimated using the following formula: Volume = d (a1 + a2 + a3 + …), where d is the distance between serial sections (200 μm) and a1, a2, a3 are DARPP-32-depleted areas for individual serial sections.

### Quantitative analysis of ataxin-3 inclusions (1H9 staining)

Images of coronal brain sections subjected to immunohistochemistry were obtained in Zeiss Axio Scan.Z1 microscope (25 μm thickness sections at 200 μm intervals). Whole-brain images were acquired with a Plan Apochromat 20 × /0.8 objective. Striatal-stained sections were selected following the same criteria for all animals: that is, the section with higher DARPP-32-depleted area in the control group was first identified and its anatomical position was considered the center for the selection of 10 sections for further 1H9-positive inclusions quantification. All striatal 1H9-positive inclusions were counted in the selected sections using automatic image analysis software (Qupath).

### Whole mouse brain processing, staining, and imaging

Whole mouse brain from a mouse-injected intrastriatal with AAV5-GFP was processed according to the SHIELD protocol [47]. The brain was cleared for several days with SmartClear II Pro (LifeCanvas Technologies), a device based on stochastic electrotransport [48]. The cleared brain was then actively immunolabeled within 24 h using SmartLabel (LifeCanvas Technologies), a device based on eFLASH technology (unpublished), which integrates stochastic electrotransport [48] and SWITCH [49]. The brain was stained with an anti-GFP antibody (1:4000; Aves Labs) along with fluorescently conjugated secondary antibodies in 1:2 (primary:secondary) molar ratios (Jackson ImmunoResearch). Next, the brain was incubated in EasyIndex (LifeCanvas Technologies) for refractive index matching and imaged at 3.6 × with SmartSPIM light sheet microscope (LifeCanvas Technologies).

### Statistical analysis

Statistical analysis was performed using Prism GraphPad software. Data are presented as mean ± standard error of mean (SEM) and outliers were removed according to Grubb's test (alpha = 0.05). One-way analysis of variance test was used for multiple comparisons. Correlations between parameters were determined according to Pearson's correlation coefficient. Significance was determined according to the following criteria: *P* > 0.05 = not significant (ns); *P < 0.05, **P < 0.01 ***P < 0.001, and ****P < 0.0001.

## Results

### AAV5 distribution and ATXN3 miRNA expression in mouse brain

**FIG. 1.**
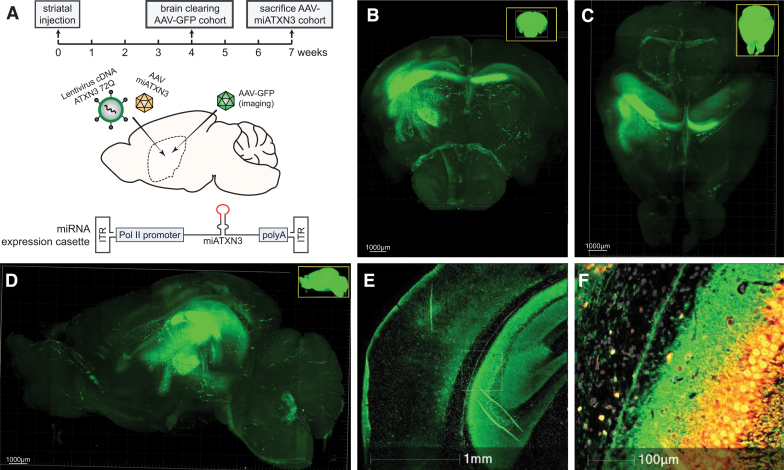
AAV injections and vector distribution after striatal injection in the mouse brain. **(A)** Timeline and overview of experimental setup. Wild-type mice were injected with 1.3 × 10^11^ gc of AAV5-GFP in 2 μL in the right striatum and killed after 4 weeks. Brains were cleared and imaged for GFP expression. A separate cohort of mice was injected bilaterally with a combination of a lentiviral construct overexpressing mutant ATXN3 cDNA and AAV5-miATXN3 to assess molecular SCA3 hallmarks. A schematic representation of the miRNA expression cassette is shown between the ITR of the AAV. A pol II promoter drives expression of the miATXN3 guide strand. **(B)** Imaging of cleared brain from GFP cohort mice. Coronal (*front*) view of brain **(C)** *Top* view **(D)** lateral *side* view of injection side **(E)** Higher magnification of hippocampus 35 days after AAV5-GFP injection. **(F)** Overlay of vector DNA probe (*red* signal) confirms GFP expression in neurons that contain the vector DNA. High levels of GFP expression were present close to the injection site (*right* striatum). Additional AAV spread to nearby cortical region and contralateral striatum was also observed. AAV, adeno-associated virus; ITR, inverted tandem repeats; GFP, green fluorescent protein. Color images available online.

To first establish whether the therapeutic AAV used here was capable of efficiently transducing the mouse striatum, a GFP expression cassette driven by the CAG promoter was packaged into AAV5 and injected unilaterally in the right striatum of wild-type mice. GFP-AAV-injected mice were killed 4 weeks after injection (Fig. 1), and brains were cleared to allow for imaging of the entire brain and accurately visualize the extent of AAV transduction by GFP expression.

A high level of GFP expression was found in the caudate putamen of the striatum close to the injection site, with a significant spread observed especially to the dorsal striatum. Localized transduction in the cortex directly above injection site was also observed, and transduction of striatal fibers and spread through corpus callosum to the contralateral hemisphere was also present. Hence it was concluded that ample AAV targeting of the affected striatal region in the LV-SCA3 mice could be achieved.

**FIG. 2.**
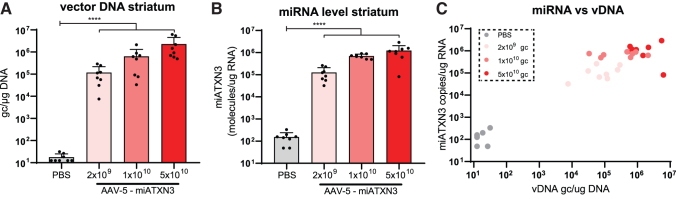
AAV transduction and miRNA expression after striatal injection. **(A)** AAV vector DNA and miRNA **(B)** expression levels in striatum of AAV-mi*ATXN3*-treated mice assessed by TaqMan probe-based qPCR analysis. The correlation between vector DNA and miRNA expression level **(C)** was confirmed. *N* = 8 mice per treatment group. Indicated dosages are in genome copies (gc) per animal. ****P < 0.0001 as tested by one-way ANOVA on log-transformed expression values. ANOVA, analysis of variance. Color images available online.

Next, to establish functional improvement at the molecular level, a previously described AAV5 carrying a miRNA expression cassette targeting ATXN3 [37] was coinjected together with a lentivirus encoding human ATXN3 cDNA with 72 CAGs bilaterally in the mouse striatum (Fig. 1A). Approximately 7 weeks after injection, presence of vector DNA (Fig. 2A) and expression of the mature miRNA (Fig. 2B) was confirmed by qPCR analysis.

A dose-dependent increase in both vector DNA, up to ∼1 × 10^7^ gc/μg DNA, and corresponding miRNA levels were observed. Expression levels were well within the effective range required for target knockdown based on previous studies [37,50–53]. Furthermore, the level of vector DNA correlated well with the expression of the miATXN3 guide strand (Fig. 2C), indicating that no plateau of miRNA expression was reached yet at the viral loads used here.

### AAV5-mi*ATXN3* induces strong ataxin-3 knockdown in a lentiviral SCA3 mouse model

To confirm *in vivo* potency of AAV5-delivered mi*ATXN3,* bilateral striatal injections were performed in wild-type mice. AAV5-mi*ATXN3* was coinjected with a lentiviral vector encoding human mutant ataxin-3 with 72Q (LV-mutATXN3). This lentiviral SCA3 mouse model presents strong expression of mutant ataxin-3 throughout the striatum, resulting in several molecular hallmarks of disease in this brain structure [44]. Previous research has shown that expression of this LV-mutATXN3 construct is stable out to at least 10 weeks postinjection [42]. By coinjecting an AAV carrying a miRNA targeting ATXN3, this model can hence be used to determine alleviation of molecular SCA3 hallmarks in a controlled manner.

**FIG. 3.**
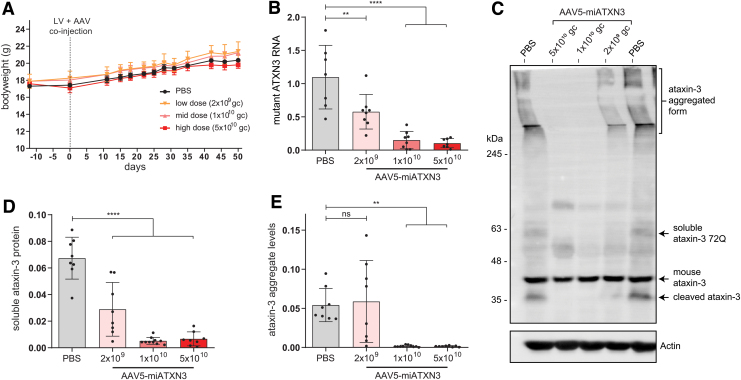
miATXN3-mediated ataxin-3 knockdown in SCA3 mouse brain. Mice were stereotaxically injected at 2 months of age with a mixture of a lentiviral vector comprising a human mutant ataxin-3 cDNA with 72 CAGs and AAV5-miATXN3 in both striata. The lentiviral construct resulted in expression of mutant ataxin-3 throughout the striatum during the study period. **(A)** Bodyweight of mice was comparable between treatment groups and was not negatively affected by any of the tested doses of AAV5-miATXN3. **(B)** qPCR analysis revealed a strong dose dependent knockdown of mutant *ATXN3* transcript expression in the striatum 7 weeks after AAV5-miATXN3 treatment. **(C)** Example western blot of SCA3 mouse striatum, showing aggregated- (high molecular weight), soluble- and cleaved-mutant ataxin-3 protein fractions. Endogenous mouse ataxin-3 protein was also observed ∼40 kDa. **(D)** Soluble ataxin-3 protein levels were reduced up to 90% in the striatum after high dose of mi*ATXN3* as quantified through western blot analysis. **(E)** The insoluble and aggregated ataxin-3 protein fraction in striatum was almost completely abolished by mid- and high-dose treatment of mi*ATXN3.* Indicated dosages are in genome copies (gc) per animal. *N* = 8 for all treatment groups. Analyzed by one-way ANOVA (**P < 0.01 and ****P < 0.0001). ns, non-significant. Color images available online.

Mice were monitored for ∼7 weeks after injection, and no effect of the AAV on bodyweight was observed during this period (Fig. 3A). The tolerability of AAV5-miATXN3 was further confirmed in a separate cohort of wild-type mice, where a total dose of 1.3 × 10^11^ gc was compared with formulation buffer when injected in striatum. No effect on bodyweight was observed during a 57-day period after injection (Supplementary Fig. S2). The right striatum of the mice was used for immunohistochemical analysis, whereas the left striatum was used for molecular analysis, where expression of the mutant ataxin-3 transcript was confirmed through qPCR in the PBS-treated control group (Fig. 3B).

In the mi*ATXN3*-treated mice, a robust knockdown of mutant ataxin-3 mRNA was observed in a dose-dependent manner. The low dose (2 × 10^9^ gc) of AAV5 resulted in ∼50% *ATXN3* mRNA knockdown, whereas the mid (1 × 10^10^ gc) and high dose (5 × 10^10^ gc) almost completely abolished *ATXN3* mRNA expression (Fig. 3B).

Of note, endogenous mouse *ATNX3* mRNA was not significantly affected by mi*ATXN3* treatment (Supplementary Fig. S1A), despite carrying only one mismatch in the target sequence. At the level of endogenous mouse ataxin-3 protein, an 18% and 22% lowering could be observed (Supplementary Fig. S1B) for the animals treated with mid- and high-dose AAV5-miATXN3, respectively. Owing to this minor extent of endogenous mouse ataxin-3 targeting, no claims can be made regarding safety of strong ataxin-3 reduction in this study. SCA3 knockout mice have however been established by others, where no overt toxicity was reported [20,21].

Similar to SCA3 patients, the mouse model used here presents with both soluble and insoluble forms of the mutant ataxin-3 protein. Through western blot analysis, these different states of the ataxin-3 protein can be investigated, as the high molecular weight aggregates do not migrate into the separating gel (Fig. 3C). As predicted by the mRNA results, a dose-dependent reduction in both the soluble (Fig. 3D) and insoluble ataxin-3 protein (Fig. 3E) was observed.

Of note, the putatively toxic ataxin-3 aggregates were completely abolished by the mid and high dose of mi*ATXN3* treatment (Fig. 3C, E). In addition, soluble ataxin-3 protein levels in striatum closely mirrored the observed mRNA levels, with low-dose treatment resulting in ∼50% reduction and the high-mi*ATXN3* dose reducing ataxin-3 protein levels by ∼90%. Together, these results suggest a strong potency of mi*ATXN3* against the *ATXN3* transcript, with only mild off-target efficacy at the dosages used here.

### Reduction in ataxin-3 inclusions

The lentiviral SCA3 mouse model used here also develops several histological features of SCA3 as a result of the continuous ataxin-3 (72Q) expression [44]. Of particular interest are the hallmark ataxin-3 inclusions [54,55] that form in the area transduced with the expression cassette. These protein inclusions only occur with longer polyglutamine repeat lengths in the ataxin-3 protein, and correlate with disease progression in these mice.

**FIG. 4.**
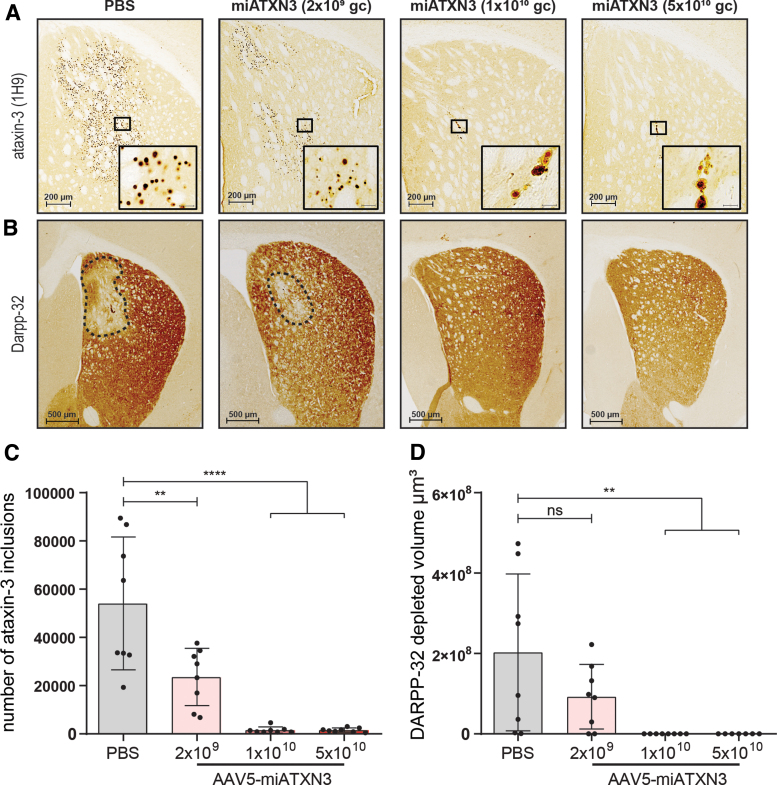
Reduction in ataxin-3 inclusions and DARPP-32 lesion size in mi*ATXN3*-treated SCA3 mice. Striatum from right hemisphere of miATXN3-treated SCA3 mice were stained for human ataxin-3 and mouse DARPP-32 protein. **(A)** Anti-ataxin-3 stained (1H9) striatum of mice killed ∼7 weeks after mi*ATXN3* treatment shows presence of nuclear inclusions in the SCA3 mice as a result of mutant ataxin-3 expression. Panels from left to right represent the different treatment groups, showing a clear reduction in nuclear ataxin-3 inclusion level with increasing dose of mi*ATXN3*. **(B)** Right striatum of mice was stained with the midbrain dopaminergic neuron marker DARPP-32. A DARPP-32-depleted lesion (*dotted outline*) representing the early neuronal dysfunction can be seen in the PBS-treated animals close to the injection site. **(C)** Quantification of nuclear ataxin-3 inclusions in striatum as shown in **(A)**. Low-dose miATXN3 treatment significantly reduced the number of ataxin-3 inclusions by ∼50%. Presence of nuclear ataxin-3 inclusions was almost completely abolished in mid- and high-dose miATXN3-treated animals. PBS *n* = 8; AAV5-miATXN3 (2 × 10^9^ gc) *n* = 8; AAV5-miATXN3 (1 × 10^10^ gc) *n* = 7; AAV5-miATXN3 (5 × 10^10^ gc) *n* = 8. Analyzed with one-way ANOVA (**P < 0.01 and ****P < 0.0001). **(D)** Quantification of DARPP-32-depleted volume. Total DARPP-32 lesion size was calculated for the whole striatum based on interspaced sections. Lesion size was significantly reduced in a dose-dependent manner after miATXN3 treatment compared with PBS-treated control animals, indicating a reduction in neuronal dysfunction. The DARPP-32 lesion was completely abolished in mid- and high-dose mi*ATXN3*-treated animals. PBS *n* = 8; AAV5-miATXN3 (2 × 10^9^ gc) *n* = 8; AAV5-miATXN3 (1 × 10^10^ gc) *n* = 8; AAV5-miATXN3 (5 × 10^10^ gc) *n* = 7. Analyzed with one-way ANOVA (**P < 0.01). ns, non-significant. Color images available online.

Similar to what was shown with the western blot analysis, histological examination of the SCA3 mouse brain revealed a robust and strong reduction in the ataxin-3 inclusion burden throughout the striatum (Fig. 4A, C). Low-dose mi*ATXN3* treatment reduced the number of ataxin-3 inclusions by ∼50% on average, whereas almost no nuclear inclusions could be detected in mid- and high-dose miATXN3-treated mice. As expected, the inclusion count in mice treated with the low-dose mi*ATXN3* (Fig. 4C) closely correlated with the 50% reduction of mutant ataxin-3 mRNA and soluble ataxin-3 protein levels in this treatment group (Fig. 3B, D), indicating a direct relation between mutant ataxin-3 expression levels and formation of the ataxin-3 inclusions.

### Rescue of neuronal dysfunction

Similar to the other polyglutamine proteins, mutant ataxin-3 is known to induce cellular stress and neuronal dysfunction over time [13,56]. We performed immunostainings on the striatal dopaminergic marker DARPP-32 to assess the extent of neuronal dysfunction in the SCA3 mice. In line with previous reports [40,44], the PBS-treated SCA3 mice presented a DARPP-32-depleted region in the striatum of ∼2 × 10^8^ μm^3^ on average (Fig. 4B, D). Low-dose mi*ATXN3* treatment resulted in an average lesion size that was half the size of PBS-treated animals, although this did not reach statistical significance (*P* = 0.19). In contrast, animals treated with mid- and high-dose mi*ATXN3* showed remarkable improvement in this phenotype, as all but one animal did not present with any observable DARPP-32-depleted area.

In early stage of polyglutamine disease, such as reported here, DARPP-32 downregulation likely represents onset of neuronal dysfunction, such as synaptic signaling deficits [57]. Moreover, DARPP-32 is involved in regulation of electrophysiological and transcriptional responses [58], further underlining the importance of retaining DARPP-32 expression to maintain neuronal health. The observed improvement in lesion size hence confirms the neuroprotective effect of mi*ATXN3-*induced knockdown of ataxin-3 in this SCA3 mouse model.

## Discussion

In this study we tested the *in vivo* functionality of a therapeutic AAV5-delivered miRNA targeting the *ATXN3* transcript as a treatment strategy for SCA3. Injection of the AAV5-miATXN3 in a striatal lentiviral-based mouse model of SCA3 resulted in strong reduction of the mutant ataxin-3 transcript and protein levels in a dose-dependent manner. Correspondingly, we observed almost complete ablation in the ataxin-3 aggregate and nuclear ataxin-3 inclusion load in the striatum of the mice, accompanied by robust reduction in DARPP-32 lesion size.

In our first tests of AAV-mi*ATNX3* in a SCA3 knock-in mouse model carrying as many as 304 mixed CAG/CAA repeats [59], injection in cisterna magna of AAV5-miATXN3 resulted in targeting of posterior fossa and ataxin-3 knockdown of ∼50% [37]. Of importance, as the CAG/CAA repeats were inserted in the endogenous ATXN3 gene in this model [59] a single nucleotide mismatch to the miATXN3 guide strand was expected. For this reason, a stronger knockdown efficacy was expected in this study.

The mouse model here used was generated by injecting a lentiviral vector expressing the full human ATXN3 cDNA sequence with 72 CAG repeats in the striatum (LV-SCA3) [44]. Although the expression of ataxin-3 was limited to the striatum, this mouse model develops robust molecular hallmarks of SCA3, including formation of polyQ containing inclusions and DARPP-32-depleted striatal lesions [40]. For this reason, the LV-SCA3 mouse is suitable to confirm target engagement and molecular proof of principle for SCA3 gene therapy approaches.

In this regard, a strong dose response was observed, with the low-dose AAV5-mi*ATXN3* (2 × 10^9^ gc per animal) achieving a roughly 50% ataxin-3 RNA and protein knockdown, and the high dose (5 × 10^10^ gc) resulting in almost complete ataxin-3 knockdown. The ataxin-3 expression levels corresponded directly with the most prominent disease hallmarks of SCA3, namely the ataxin-3 cellular inclusions and insoluble ataxin-3 protein fractions, which were also reduced to approximately half of untreated levels. Similarly, histological examination revealed a comparable reduction in DARPP-32 lesion size, indicating the relation between mutant ataxin-3 levels and neuronal health.

Owing to the simultaneous treatment window of mutant ataxin-3 expression by the lentiviral construct and downregulation achieved by the AAV5-delivered miRNA, it is not possible to directly extrapolate whether intracellular ataxin-3 aggregates that are already present can be alleviated by inhibiting ataxin-3 expression. It should be noted however that lentiviral expression usually occurs faster than that of AAV, so the actual expression of the mutant ataxin-3 protein most likely did precede therapeutic expression levels of the miRNA. Previous studies in an inducible SCA3 mouse model have shown that symptom reversal is possible when halting expression of mutant ataxin-3 in already symptomatic mice [60].

It must also be mentioned that the ataxin-3 expression level in the lentiviral mouse model used here is at least 4 × higher than the endogenous ataxin-3 [40,44]. Hence, at endogenous expression levels, a less substantial knockdown than reported here could already be sufficient to prevent onset of the nuclear ataxin-3 inclusions. Hence, future experiments with different treatment windows of AAV5-miATXN3 are useful to better assess the potential of gene therapy approaches to rescue neuronal dysfunction and ataxin-3 aggregates after onset.

The miRNA tested here has previously been designed and optimized in cell-culture experiments to induce potent ataxin-3 downregulation [37]. The selected miRNA is expressed from the miR-451 scaffold, which has the major advantage of being processed by ago2 and independently of dicer. This results in expression of only the *ATXN3*-targeting guide strand, in absence of a passenger strand that may affect off-target genes [61,62]. As tested previously in our laboratory in the context of the huntingtin gene, miR-451-based miRNAs can result in potent gene knockdown [50,51,53,63], with minimal targeting of potential off-target genes [52].

In addition, analysis of miRNA expression in neurons after treatment with miATXN3 showed no changes in endogenous miRNA levels [37]. This is important, as high levels of artificial miRNA expression can interfere with the RNAi machinery, such as ago2 processing [64], consequently dysregulating expression of naturally occurring miRNAs. The miRNA scaffold and promoter combination used here can thus potentially be used in a broad window of therapeutic dosages, allowing for controlled gene knockdown with a favorable safety profile. The first Huntington patients have recently been dosed using a similar miRNA expression cassette in a phase 1/2 clinical trial.

Similar to Huntington's disease, downregulation of the mutant transcript is regarded as a promising treatment strategy for SCA3, as the *ATXN3* transcript is the most upstream target in the pathological cascade underlying the disease [13,65].

There are currently several preclinical strategies for treatment of SCA3 under investigation, including a range of pharmacological compounds and RNA-targeting strategies (reviewed in [1]). Especially targeting ATXN3 RNA for downregulation or modulation by ASOs (reviewed in [66]) or RNAi [30,37] are currently regarded as very promising treatment strategies. Both strategies have shown both preclinical and early clinical success in central nervous system applications, with specific advantages for each approach [67,68]. An advantage of the AAV-delivered miRNA or shRNA is that a one-time treatment provides longer lasting effects over ASOs, with over 8 years of stable expression reported so far [69].

Given this one-time treatment however, targeting of the SCA3-relevant brain regions after one injection is key. In earlier rodent studies with AAV-mi*ATXN3,* we showed that injection in cisterna magna provided the most efficient targeting of brain stem and cerebellum [37]. The cisterna magna dosing technique has proven successful in larger animals as well [70], and a next important step will be to confirm adequate transduction by AAV5 of the posterior fossa in these larger animals.

It must be mentioned that the miRNA used here was not designed to induce allele-specific knockdown. Although the current evidence indicates that ataxin-3 knockout is well tolerated in rodents, several functions such as deubiquitination [15], transcriptional regulation [71], and involvement in the DNA damage response [23] are ascribed to the protein. For this reason, complete knockdown of ataxin-3 within the cell may not be the most suitable goal for an ultimate gene therapy approach for SCA3.

Instead, achieving sufficient ataxin-3 knockdown to prevent onset of cellular toxicity may be a more desirable strategy. Allele-specific shRNAs have also been tested for SCA3 [28,33,42,72], which would improve safety in this regard, but on the contrary, would exclude ∼30% of the SCA3 patient population owing to absence of the relevant single nucleotide polymorphism [73]. For this reason, future studies in large animal models examining efficacy and safety of mi*ATXN3*-induced ataxin-3 knockdown are required.

To summarize, here we have shown the efficacy of an *ATXN3*-targeting miRNA to induce ataxin-3 knockdown in a mouse model for SCA3. A robust rescue of the molecular SCA3 hallmarks was observed, namely reduction in ataxin-3 aggregates and DARPP-32 lesion size in brain tissue. These studies add to an earlier body of work showing efficacy of AAV5-mi*ATXN3* in human-induced pluripotent stem cell-derived neurons, mouse models, and minipigs [37], providing a good base to further pursue development of a gene therapy-based therapeutic approach for SCA3.

## Supplementary Material

Supplemental data

Supplemental data
